# Commentary: Diagnostic accuracy of oral swab for detection of pulmonary tuberculosis: a systematic review and meta-analysis

**DOI:** 10.3389/fmed.2025.1568093

**Published:** 2025-07-07

**Authors:** Neto Pascoal, Bista Florindo Luís Caetano, Nashon D. Majaliwa, José M. Viriato, Israel C. Avelino, Luzia Gonçalves

**Affiliations:** ^1^Faculty of Science and Technology, Zambeze University, Beira, Mozambique; ^2^Center for Statistics and Applications of the University of Lisbon (CEAUL), Lisbon, Portugal; ^3^z-Stat4life, Lisbon, Portugal; ^4^Instituto Nacional de Saúde, Vila de Marracuene, Maputo, Mozambique; ^5^Department of Microbiology, Faculty of Medicine, Eduardo Mondlane University, Maputo, Mozambique; ^6^Department of Public Health and Infectious Diseases, Sapienza University of Rome, Rome, Italy; ^7^Pathogen Genome Bioinformatics and Computational Biology, Faculty of Pharmacy, Research Institute for Medicines (iMed-ULisboa), University of Lisbon, Lisbon, Portugal; ^8^Center for Advanced Studies in Medical Education and Training, Faculty of Medicine, Agostinho Neto University, Luanda, Angola; ^9^Medical Surgical Research Center of Angola-Multiperfil Clinic, Luanda, Angola; ^10^Global Health and Tropical Medicine, LA-REAL, Institute of Hygiene and Tropical Medicine, NOVA University of Lisbon, Lisbon, Portugal

**Keywords:** tuberculosis, oral swab, publication bias, meta-analysis, *metafor* and *meta*

## Introduction

Pulmonary tuberculosis caused by *Mycobacterium tuberculosis* remains a major public health concern, requiring accurate diagnosis for effective treatment, prevention, and transmission control, particularly in vulnerable populations. Among available diagnostic approaches, oral swab is regarded as a promising non-invasive and alternative test, especially in cases where sputum collection is difficult. Thus, many studies have explored its potential in managing this disease (1–3).

While analyzing the paper published by Zhang et al., *Diagnostic Accuracy of Oral Swab for Detection of Pulmonary Tuberculosis: A Systematic Review and Meta-Analysis* (https://doi.org/10.3389/fmed.2023.1278716), we found that the paper provides valuable insights on the topic; however, we identified an error that requires correction and felt it necessary to bring this to your attention.

To evaluate publication bias, we replicated the meta-analysis using R software (4), employing the *metafor* and *meta* packages. The analysis was conducted using the same dataset as Zhang et al. (1), which comprised 16 studies including both adult and paediatric populations. A key advantage of the *metafor* and *meta* packages is their capacity to provide separate assessments of publication bias for sensitivity and specificity. This stands in contrast to Deeks' test, which examines bias collectively through the diagnostic odds ratio (DOR).

Deeks' test, as described by Deeks, Macaskill, and Irwig (5), relies on a weighted linear regression of the logarithm of the odds ratio (logOR) on the inverse square root of the sample size (1/√n). When zero values are present in the 2 × 2 contingency table, the resulting estimates may become infinite, requiring a continuity correction by adding 0.5 to each cell (6). Within this framework, publication bias is indicated if *P* < 0.10.

This commentary therefore focuses on evaluating the diagnostic accuracy of the test, with particular attention to the influence of the identified publication bias.

## Subsections relevant for the subject

In the Results section, page 4, subsection “Publication bias assessment”, the statement “*The funnel plot clearly indicated the absence of significant publication bias in this meta-analysis (P* = *0.99)”* is inaccurate. Given the z-Stat4life community's interest in this topic, we replicated the meta-analysis using R Program (*metafor* and *meta*) (7, 8) and we found indications of the publication bias in the funnel plots and Egger test. For *metafor*, in adults, the P-values were 0.001 both for sensitivity (Se) and specificity (Sp) and for *meta* (Se: *P* = 0.007; Sp: *P* = 0.015), contrary to the paper's findings.

In the aggregate data, the results for the *meta* package showed a non-significant publication bias for sensitivity (Se: *P* = 0.054), while the *metafor* package yielded a similar outcome (Se: *P* = 0.068). These findings could be impacted by substantial residual heterogeneity (τ^2^ ≈ 5.86). For Sp, however, a statistically significant publication bias was detected (*P* = 0.001), with comparatively lower residual heterogeneity (τ^2^ ≈ 2.14) across both *meta* and *metafor*.

Deeks' test indicated *P* = 0.054, suggesting presence of publication bias (*p* < 0.10). This was visually apparent in the funnel plots and was further supported by Egger's test. Additionally, significant asymmetry was identified using Deeks' test in both the adult-specific dataset and the combined dataset (adults and children).

## Discussion and final considerations

Publication bias affecting both Se and Sp in this meta-analysis may inflate the test's diagnostic accuracy, potentially misleading clinical decisions. Although the analyses consistently indicated the presence of publication bias in adults, the distinctive methodological characteristics of various approaches may result in divergent results depending on the software packages and analytical techniques used. For this reason, the use of complementary analytical strategies is strongly recommended to support a critical and comprehensive evaluation of the findings.

Given the importance of this topic, it is crucial to perform subgroup analyses while taking the following points into consideration:

- The selection of statistical software and modeling strategies can substantially affect the results, particularly in scenarios involving high heterogeneity or limited sample sizes;
- Ensuring the reproducibility and reliability of diagnostic meta-analyses requires not only the implementation of diverse methods for detecting publication bias but also a deep understanding of the underlying assumptions and limitations of each approach;
- Clear and transparent reporting of the analytical code, along with any modifications applied (such as continuity corrections), is vital for enabling reproducibility and facilitating cross-study comparisons;
- Comprehensive disclosure of all results, including non-significant or negative findings combined with a strong commitment to methodological rigor, is essential to reducing bias and strengthening the credibility of scientific evidence.

Therefore, attention to these issues and appropriate editorial actions are essential to maintain the journal's scientific quality and credibility.
