# Supplementary material for: Commentary: Diagnostic accuracy of oral swab for detection of pulmonary tuberculosis: a systematic review and meta-analysis
Source: Front Med (Lausanne). 2025 Jul 7;12:1568093. doi: 10.3389/fmed.2025.1568093 (PMC12277163; doi:10.3389/fmed.2025.1568093)
Supplement: Supplementary file 1 [file Data_Sheet_1.docx]

**SUPPLEMENTARY MATERIAL**

**1. ASSESSMENT OF PUBLICATION BIAS**

This section provides a detailed description of the procedure adopted for the assessment of publication bias, based on both the Adult Data (considered in our manuscript) and the Aggregated Data (adults and children) to better understand the differences in results of the two manuscripts.

The exposition is organized into two main parts — one referring to the adult data and the other to the aggregated data — each subdivided into three subsections: (i) results obtained using the *metafor* package, (ii) results obtained using the *meta* package, and (iii) application of Deeks’ test.

For conducting statistical analysis in R, it is essential to ensure that the *meta* and *metafor* packages are properly installed using the *install.packages() function*, and subsequently loaded into the workspace using the commands *library(meta)* and *library(metafor)*.

**Table 1**. Aggregated Study Data (Adults and Children) – from Zhang et al. 2024


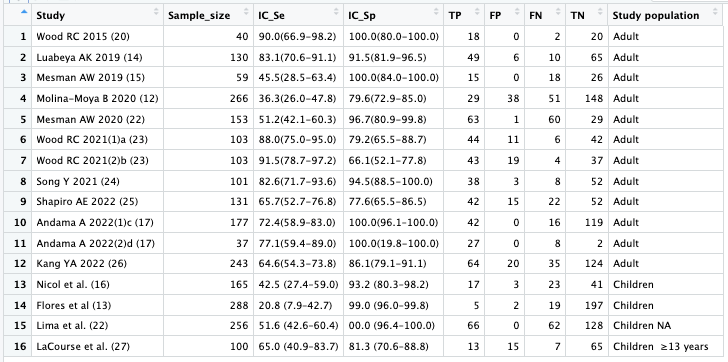


**2. ASSESSMENT OF PUBLICATION BIAS IN STUDIES WITH ADULT DATA**

**2.1. RESULTS USING THE *METAFOR* PACKAGE: EGGER'S TEST, FUNNEL PLOTS, AND TESTS FOR SENSITIVITY AND SPECIFICITY**

Below is the script for the *metafor* package:

data <- aggregated_data

data <- data[, -c(2, 3, 4, 9)] # Remove variables 2, 3, 4 and 9

data <- data[1:12, ]

# Before, it was necessary to apply a continuity adjustment to avoid zero values.

data$Adjusted_TP <- data$TP + 1

data$Adjusted_FN <- data$FN + 1

data$Adjusted_FP <- data$FP + 1

data$Adjusted_TN <- data$TN + 1

data$Sensitivity <- data$Adjusted_TP / (data$Adjusted_TP + data$Adjusted_FN)

data$Specificity <- data$Adjusted_TN / (data$Adjusted_TN + data$Adjusted_FP)

data$Sens_Logit <- log(data$Sensitivity / (1 - data$Sensitivity))

data$Spec_Logit <- log(data$Specificity / (1 - data$Specificity))

data$Sens_SE <- sqrt((1 / data$Adjusted_TP) + (1 / data$Adjusted_FN))

data$Spec_SE <- sqrt((1 / data$Adjusted_TN) + (1 / data$Adjusted_FP))

data$Sens_SE <- pmax(data$Sens_SE, 0.01)

data$Spec_SE <- pmax(data$Spec_SE, 0.01)

meta_sens <- rma(yi = data$Sens_Logit, sei = data$Sens_SE, data = data, method = "REML")

meta_spec <- rma(yi = data$Spec_Logit, sei = data$Spec_SE, data = data, method = "REML")

funnel(meta_sens, main = "Funnel Plot for Sensitivity")

funnel(meta_spec, main = "Funnel Plot for Specificity")

egger_sens <- regtest(meta_sens, model = "rma", predictor = "sei")

print(egger_sens)

egger_spec <- regtest(meta_spec, model = "rma", predictor = "sei")

print(egger_spec)

**Output: Sensitivity Estimates Obtained Using the *metafor* Package**

> egger_sens <- regtest(meta_sens, model = "rma", predictor = "sei")

> print(egger_sens)

Regression Test for Funnel Plot Asymmetry

Model: mixed-effects meta-regression model

Predictor: standard error

Test for Funnel Plot Asymmetry: z = 3.3275, p = 0.0009

Limit Estimate (as sei -> 0): b = 0.7975 (CI: 0.1523, 1.4427)


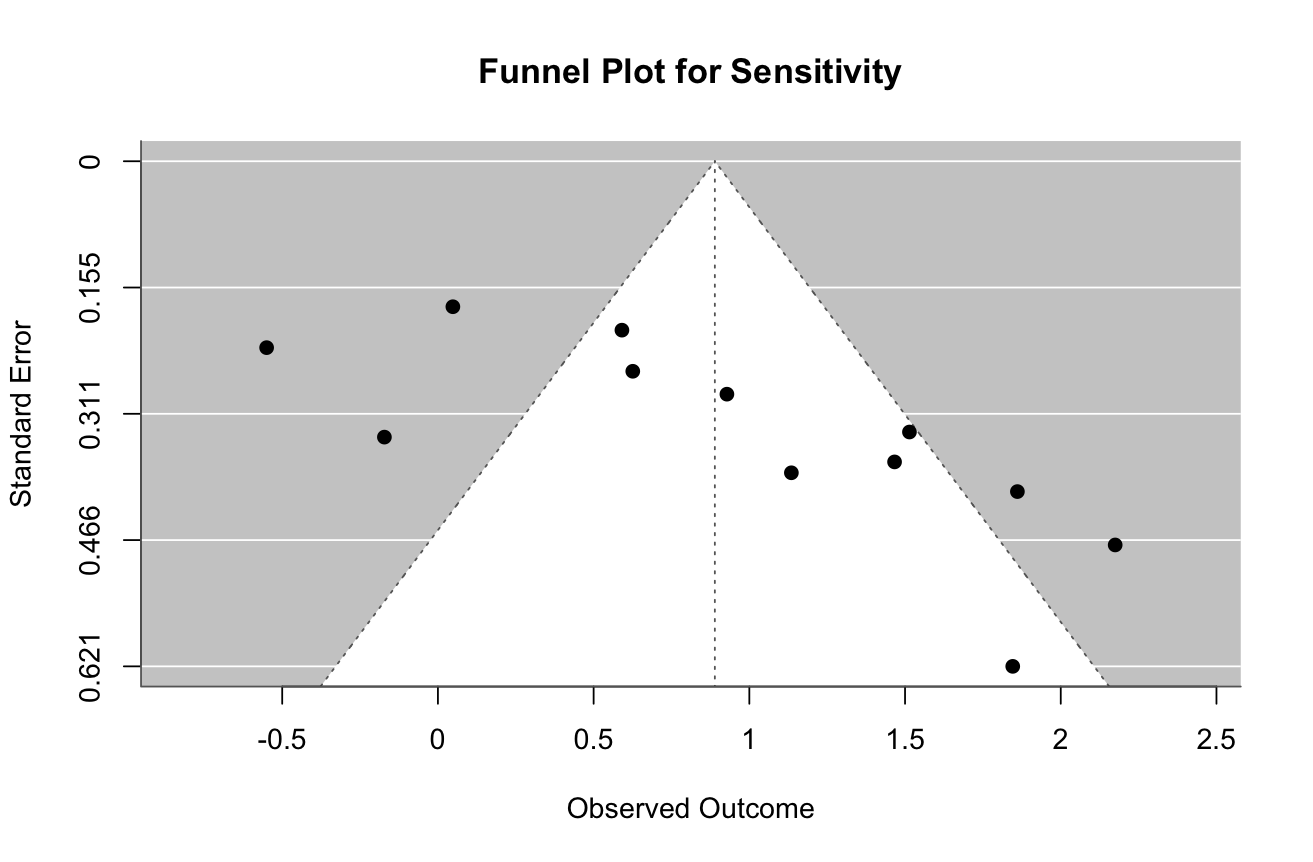


**Fig.1** – Sensitivity Funnel Plot for adult data

**Output: Specificity Estimates Obtained Using the *metafor* Package**

> egger_spec <- regtest(meta_spec, model = "rma", predictor = "sei")

> print(egger_spec)

Regression Test for Funnel Plot Asymmetry

Model: mixed-effects meta-regression model

Predictor: standard error

Test for Funnel Plot Asymmetry: z = 3.3275, p = 0.0009

Limit Estimate (as sei -> 0): b = 0.7975 (CI: 0.1523, 1.4427)


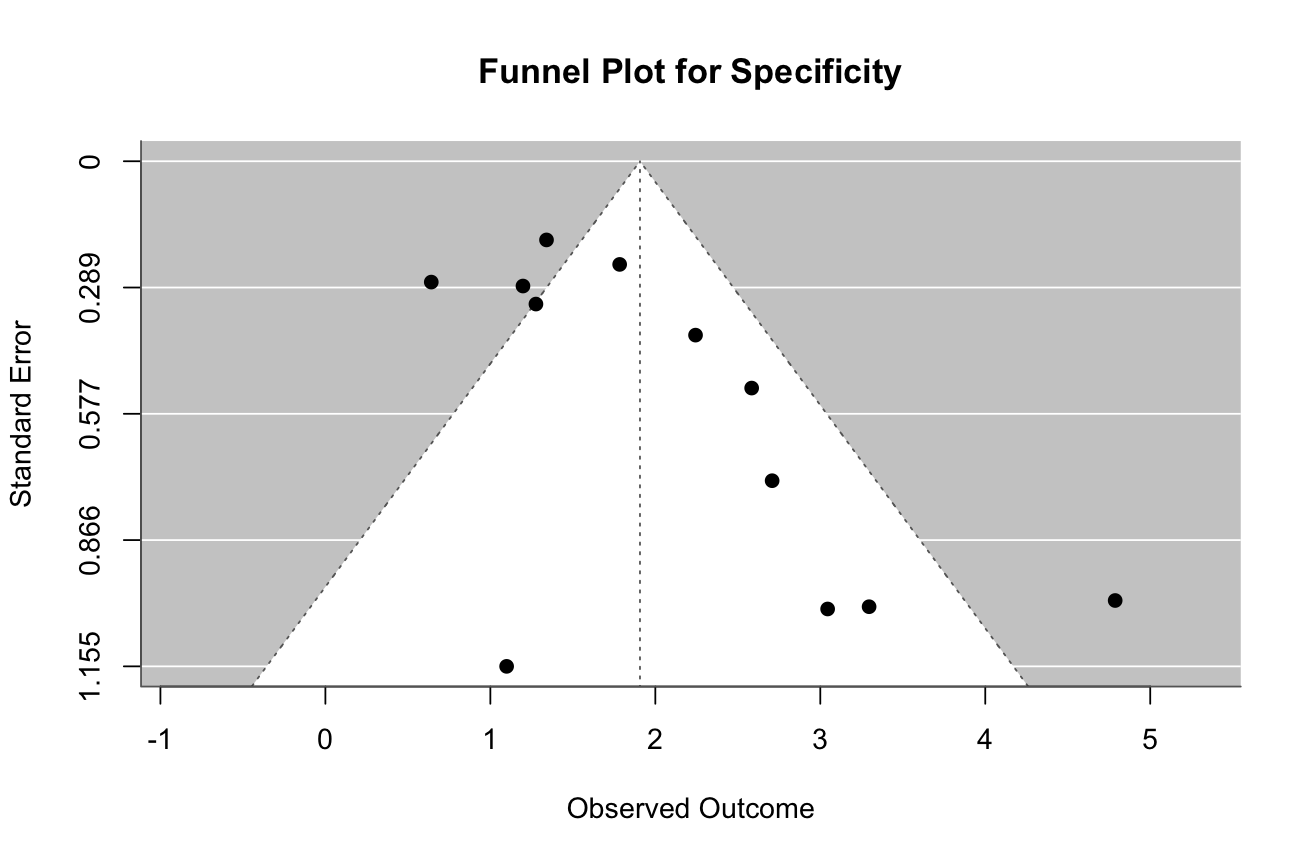


**Fig. 2** – Specificity Funnel Plot for adult data

**2.2**. **RESULTS USING THE *META* PACKAGE: EGGER'S TEST, FUNNEL PLOTS, AND TESTS FOR SENSITIVITY AND SPECIFICITY**

**Below is the script for the *meta* package:**

data <- aggregated_data

data <- data[, -c(2, 3, 4, 9)] # Remove variables 2, 3, 4 and 9

data <- data[1:12, ] # Retention of the 12 studies with adults

meta_sens <- metaprop(event = data$TP, n = data$TP + data$FN,

studlab = data$Study, sm = "PLOGIT", method = "GLMM")

meta_spec <- metaprop(event = data$TN, n = data$TN + data$FP,

studlab = data$Study, sm = "PLOGIT", method = "GLMM")

summary(meta_sens)

summary(meta_spec)

funnel(meta_sens, main = "Funnel plot for Sensitivity")

funnel(meta_spec, main = "Funnel plot for Specificity")

metabias(meta_sens, method.bias = "linreg")

metabias(meta_spec, method.bias = "linreg")

**Output: Sensitivity Estimates Obtained Using the *meta* Package**

Teste de Egger para sensibilidade

> metabias(meta_sens, method.bias = "linreg")

Linear regression test of funnel plot asymmetry

Test result: t = 3.40, df = 10, p-value = 0.0067

Bias estimate: 5.5825 (SE = 1.6398)

Details:

- multiplicative residual heterogeneity variance (tau^2 = 3.8777)


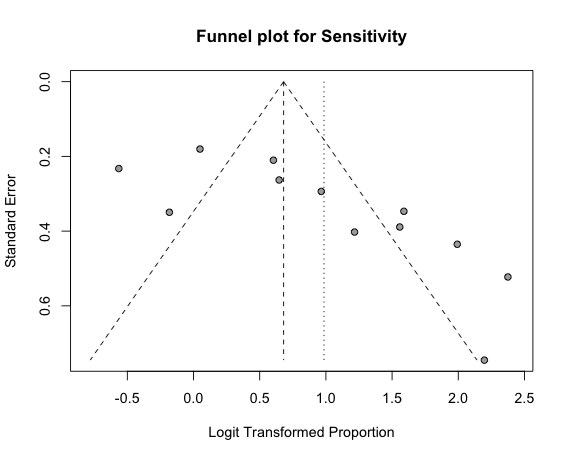


**Fig. 3** – Sensitivity Funnel Plot for aggregated data

Egger’s test applied to the specificity parameter revealed significant publication bias (t = 2.91; df = 10; p = 0.0154), with a bias estimate of 2.14 (SE = 0.73). The moderate residual heterogeneity (τ² = 2.07) should be considered when interpreting the results.

**Output: Specificity Estimates Obtained Using the *meta* Package**

Egger's test for specificity

> metabias(meta_spec, method.bias = "linreg")

> metabias(meta_spec, method.bias = "linreg")

Linear regression test of funnel plot asymmetry

Test result: t = 2.91, df = 10, p-value = 0.0154

Bias estimate: 2.1352 (SE = 0.7325)

Details:

- multiplicative residual heterogeneity variance (tau^2 = 2.0657)


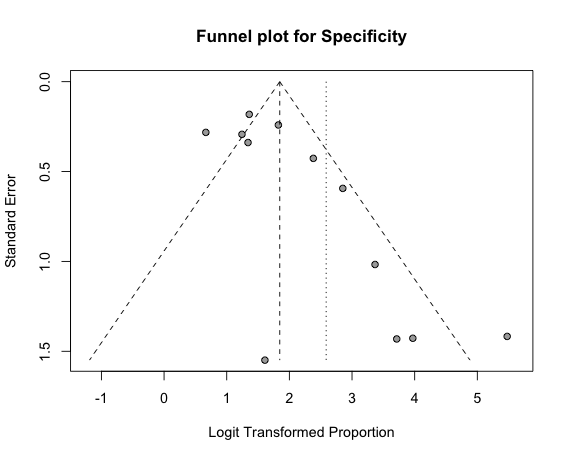


**Fig. 4** – Specificity Funnel Plot for aggregated data

**Summary on Egger's test using the *meta* package:** The test indicated significant publication bias for both sensitivity (p = 0.0067; bias = 5.58, SE = 1.64; τ² = 3.88) and specificity (p = 0.0154; bias = 2.14, SE = 0.73; τ² = 2.07). The moderate residual heterogeneity in both parameters should be considered in the interpretation of the results.

**2.3. APPLICATION OF THE DEEKS TEST**

In the same dataset from Table 1, a continuity correction was applied.

data$logOR <- log((data$TP * data$TN) / (data$FP * data$FN))

data$var_logOR <- 1/data$TP + 1/data$FP + 1/data$FN + 1/data$TN

data$inv_sqrt_n <- 1 / sqrt(data$TP + data$FN + data$FP + data$TN)

deeks_model <- lm(logOR ~ inv_sqrt_n, weights = 1/var_logOR, data = data)

coef_names <- names(coef(deeks_model))

names(deeks_model$coefficients) <- gsub("inv_sqrt_n", "Bias", coef_names)

names(deeks_model$terms) <- gsub("inv_sqrt_n", "Bias", names(deeks_model$terms))

deeks_model$call$formula <- as.formula("logOR ~ Bias")

summary(deeks_model)

**Output: Deeks test estimates**


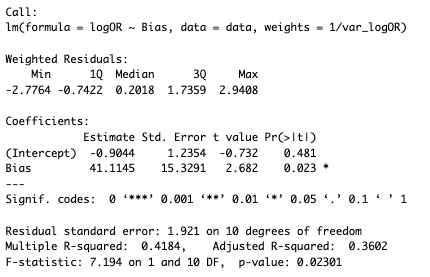


**Fig. 5** – Deeks test output for aggregated data

Deeks’ test revealed a statistically significant association between bias and the logOR, with a p-value of 0.02301. This result provides clear evidence of asymmetry in the funnel plot, suggesting the presence of publication bias in the meta-analysis, in accordance with the criterion established by Deeks et al. (2005), which considers p-values below 0.10 indicative of publication bias.

**3. EVALUATION OF PUBLICATION BIAS WITH AGGREGATED DATA (ADULTS AND CHILDREN)**

In this section, the same packages and scripts used for the assessment of bias in the Adult Data are applied, now adapted to the Aggregated Data (adults and children) presented in Table 1. For this purpose, the set of 16 studies is maintained, as indicated in the code below:

data <- data <- aggregated_data

data <- data[, -c(2, 3, 4, 9)]

**3.1. RESULTS USING THE *METAFOR* PACKAGE: EGGER’S TEST, FUNNEL PLOTS, AND TESTS FOR SENSITIVITY AND SPECIFICITY**

**Output: Sensitivity estimates obtained with the *metafor* package**

> print(egger_sens)

Regression Test for Funnel Plot Asymmetry

Model: mixed-effects meta-regression model

Predictor: standard error

Test for Funnel Plot Asymmetry: z = 1.8284, p = 0.0675

Limit Estimate (as sei -> 0): b = -0.5115 (CI: -1.7931, 0.7700)


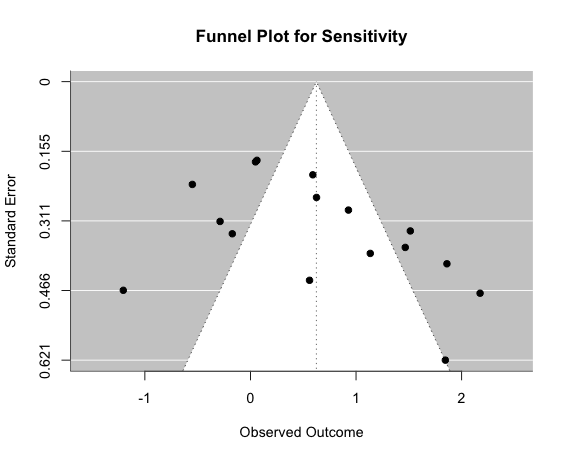


**Fig. 6** – Sensitivity Funnel Plot for aggregated data

**Output: Specificity Estimates Obtained Using the *metafor* Package**

> print(egger_spec)

Regression Test for Funnel Plot Asymmetry

Model: mixed-effects meta-regression model

Predictor: standard error

Test for Funnel Plot Asymmetry: z = 4.3865, p < .0001

Limit Estimate (as sei -> 0): b = 0.7024 (CI: 0.0386, 1.3661)


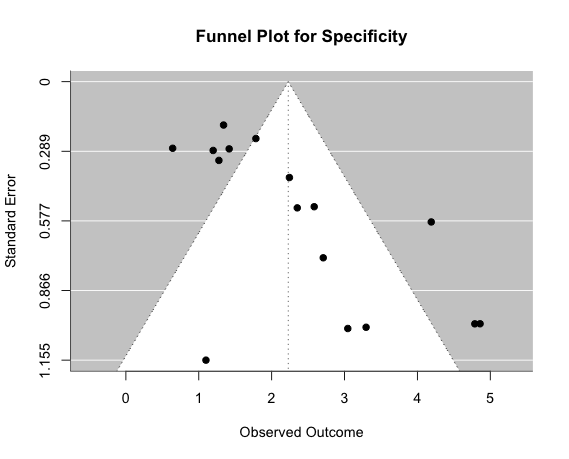


**Fig. 7** – Specificity Funnel Plot for aggregated data

**3.2. RESULTS WITH THE META PACKAGE: EGGER TEST, FUNNEL CHARTS AND TESTS FOR SENSITIVITY AND SPECIFICITY**

**Output: Sensitivity estimates obtained with the meta package**

> metabias(meta_sens, method.bias = "linreg")

Linear regression test of funnel plot asymmetry

Test result: t = 2.11, df = 14, p-value = 0.0536

Bias estimate: 3.4838 (SE = 1.6535)


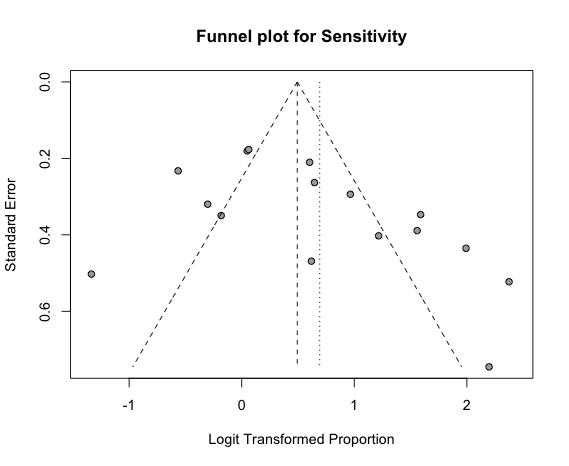


**Fig. 8** – Sensitivity Funnel Plot for aggregated data

Egger’s test for sensitivity yielded a p-value of 0.0536, suggesting signs of asymmetry in the funnel plot. The bias estimate was 3.48 (SE = 1.65), with high residual heterogeneity (τ² = 5.8610), which may influence the interpretation of potential publication bias.

**Output: Specificity estimates obtained with the meta package**

> metabias(meta_spec, method.bias = "linreg")

Linear regression test of funnel plot asymmetry

Test result: t = 4.18, df = 14, p-value = 0.0009

Bias estimate: 2.7288 (SE = 0.6535)


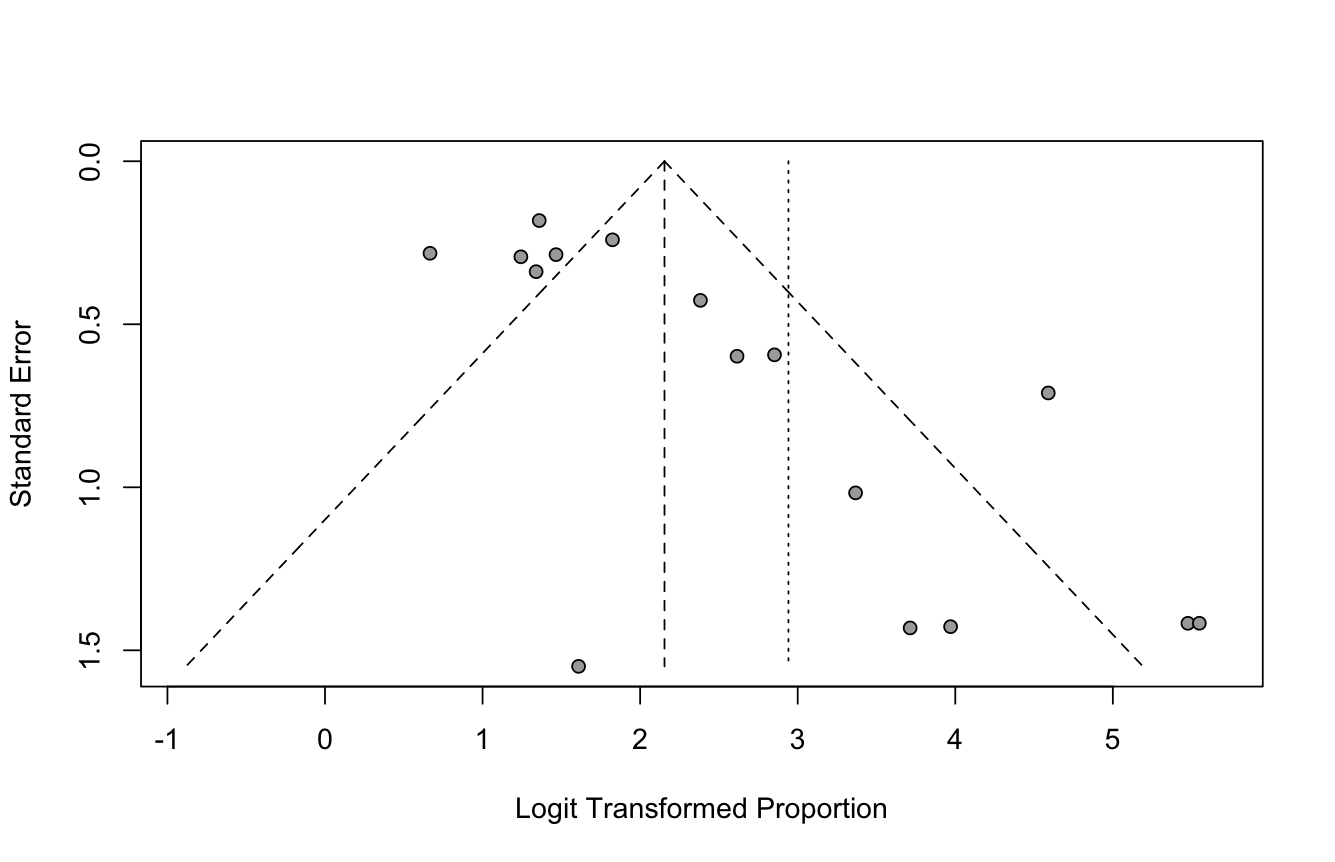


**Fig. 9** – Specificity Funnel Plot for aggregated data

**3.3. APPLICATION OF THE DEEKS TEST**

In the same dataset from Table 1, a continuity correction was applied.

data <- data <- aggregated_data

data <- data[, -c(2, 3, 4, 9)]

**Output: Deeks Test estimates**


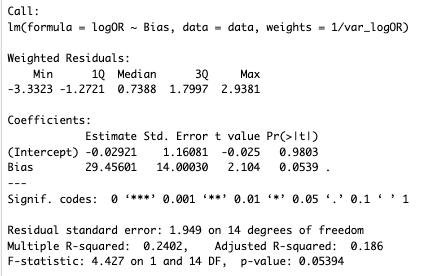


**Fig. 10:** – Deeks test output for aggregated data
